# Informal Digital Coordination of Nurse Shift Swaps in a Saudi Tertiary Hospital: A Qualitative Descriptive Study

**DOI:** 10.3390/healthcare14182957

**Published:** 2026-09-10

**Authors:** Thurayya Eid, Bader M. Almutairy, Abdulaziz M. Alodhailah, Mohammed Almutairi, Waleed M. Alshehri

**Affiliations:** 1 Department of Medical-Surgical Nursing, College of Nursing, King Saud University, Riyadh 11451, Saudi Arabiawalshehri1@ksu.edu.sa (W.M.A.); 2 Department of Community and Psychiatric Mental Health Nursing, College of Nursing, King Saud University, Riyadh 11451, Saudi Arabia

**Keywords:** nurse scheduling, shift work, digital communication, workforce management, reciprocity, electronic rostering, qualitative research, Saudi Arabia

## Abstract

Background/Objectives: Nurses may use digital communication channels alongside formal rostering systems to negotiate shift swaps. How these practices are organized, governed, and experienced in Saudi hospitals remains insufficiently understood. This study explored how nurses in one tertiary hospital coordinated shift swaps through digital communication and navigated reciprocity, professional accountability, and access to scheduling flexibility. Methods: A qualitative descriptive study was conducted in a tertiary hospital in the Riyadh region, Saudi Arabia. Twenty-four registered nurses, recruited by purposive maximum-variation sampling from medical–surgical, intensive care, emergency, and specialty units, completed individual semi-structured interviews incorporating digital journey-mapping prompts. Interviews were audio-recorded, transcribed, de-identified, and analyzed using reflexive thematic analysis, reported with reference to the Reflexive Thematic Analysis Reporting Guidelines. Results: Four interrelated themes were developed: (1) digital pathways of negotiation; (2) reciprocity as social currency; (3) managing risk and responsibility; and (4) collective coping and unequal flexibility. Private and group messaging supported initial negotiation, whereas managerial approval and formal roster documentation established legitimacy and accountability. Reciprocal assistance increased flexibility but could also generate obligation and reproduce inequalities associated with seniority, trust, and social integration. Conclusions: In this hospital, digital shift-swap coordination operated alongside, rather than in place of, formal scheduling governance. Transparent procedures connecting peer communication with managerial approval may support flexibility while protecting fairness, documentation, professional boundaries, and appropriate staffing. These findings describe one purposively recruited sample in a single hospital and require evaluation in multi-center research.

## 1. Introduction

Hospital nursing requires continuous staffing across day, night, weekend, and holiday shifts. Rostering processes must balance service requirements, patient acuity, workload, staff competence, fatigue, and nurses’ personal circumstances. Long or poorly configured shifts may increase fatigue and occupational risk, while nurses’ preferences for particular shift patterns are influenced by health, family responsibilities, commuting, and work–life balance [1,2]. Existing rostering processes can provide organizational structure but may also have limitations related to flexibility, fairness, and fatigue management [3].

A shift swap is an arrangement in which nurses exchange assigned shifts, normally subject to managerial approval and formal roster documentation. It differs from broader schedule changes, which may include leave, overtime, reassignment, or management-initiated alterations. In this study, informal digital coordination refers to peer-to-peer communication through private messages, unit messaging groups, or internal communication channels before or alongside formal approval and roster updating.

The coordination of healthcare work frequently crosses professional, organizational, and technological boundaries [4]. Digital technologies may support rapid communication and managerial processes, but their implementation depends on user acceptance, organizational arrangements, digital infrastructure, and locally established practices [5,6,7,8]. Digital platforms can bring together multiple users and activities, yet their value is shaped by governance, access, and the relationships among platform participants [6]. Digitalization is also a temporal process: technologies can change when and how work occurs, accelerate communication, and extend work-related interactions beyond conventional working hours [9].

Informal messaging may allow nurses to identify available colleagues, discuss requests discreetly, and respond rapidly to unexpected circumstances. Smartphone communication and secure messaging are increasingly integrated into clinicians’ work, although their use raises concerns about privacy, interruption, documentation, role boundaries, and expectations of availability [10,11].

These concerns are documented rather than hypothetical, and they bear directly on how scheduling requests travel. A survey of 283 hospital physicians and nurses found professional use of a consumer messaging application to be almost universal, perceived organizational guidance to be minimal, and nurses to report higher perceived risk than physicians; the authors characterized this gap between practice and policy as routine non-compliance becoming normalized [12]. A qualitative study of frontline health workers reported a comparable pattern, in which a consumer messaging platform became the de facto coordination channel while producing information overload, blurred boundaries between work and personal time, duplicated reporting across informal and official systems, and disadvantage for staff who were less digitally connected [13]. Digital professionalism is therefore relevant not only to public social media behavior, but also to the ways healthcare workers communicate and maintain professional boundaries through digital channels [14].

Digital communication does not replace organizational governance. Proposed swaps may require assessment of competence, skill mix, fatigue, workload, and staffing requirements. Official documentation remains important because agreement through messaging does not necessarily constitute an authorized schedule change. Electronic systems may themselves present usability and adoption barriers [8], while AI-supported scheduling systems introduce additional questions about fairness, transparency, and work–life balance [15].

Reciprocity and workplace social capital provide useful concepts for interpreting shift-swap practices. Workplace social capital refers to relational resources made available through trust, networks, shared expectations, and social connections [16]. Professional solidarity has similarly been associated with mutual responsibility and collegial support among nurses [17]. Nevertheless, reciprocal assistance should not automatically be classified as solidarity. Reciprocity may facilitate collective coping while also producing pressure, obligation, exclusion, or unequal access to workplace resources. Power is relational and reciprocal, although its operation in nurse–patient relationships [18] differs from the collegial relationships examined in this study.

Interprofessional collaboration has been associated with nurses’ work environments, job satisfaction, and perceived patient-safety outcomes [19]. Shared and collaborative leadership may also influence how healthcare teams coordinate under changing conditions [20]. Conversely, hierarchical cultures and perceptions that speaking up is futile may inhibit communication [21,22]. These dynamics are relevant to shift swapping because nurses may need to refuse requests, disclose personal circumstances, raise concerns about competence, or seek managerial approval.

Shift swapping may provide a practical response to illness, caregiving, examinations, religious commitments, and other changing circumstances. Similar informal practices have been described as “filling the holes” between formal workplace policies and employees’ everyday work–family needs [23]. However, successful peer coordination can conceal the organizational work required to maintain staffing. It should not be assumed to prevent burnout or resolve workload problems, which require broader organizational responses [24].

Saudi Arabia’s healthcare transformation has emphasized digitalization, service redesign, workforce development, and new models of care under Vision 2030 [25,26]. Saudi nursing workforces are professionally, culturally, and linguistically diverse. Previous studies have identified challenges associated with professional identity, cultural expectations, job retention, and workplace relationships among nurses in Saudi Arabia [27,28]. These contextual factors may influence how nurses communicate, seek assistance, and navigate hierarchy, but they should not be interpreted as fixed characteristics of Saudi nursing culture.

Existing research has examined rostering, scheduling preferences, staffing, missed care, fatigue, and workforce outcomes [1,2,3,29]. It has established that nurses value predictability and some control over working time, and that scheduling choice interacts with fatigue, well-being, and turnover intention [2,3]. Recent qualitative work has shown how the priorities of nursing staff, unit managers, and senior leaders collide during roster construction, and how collaborative or compromising responses contain that conflict while rigid ones escalate it [30]. Research has also considered coordination across the healthcare and social-service boundaries [4,31].

What is less well-described is the individual shift swap as an object of study in its own right. Rostering research generally treats the roster as the unit of analysis and the swap as an administrative adjustment within it, so three things remain largely unexamined. First, how a swap is initiated and negotiated between peers before it reaches a manager. Second, how that negotiation is conducted through informal digital channels that sit outside the electronic rostering system and outside its audit trail. Third, how the flexibility produced by these arrangements is distributed among nurses who differ in seniority, tenure, language, and social connection. This study addressed those three questions.

This study therefore addressed the following research question:

How do nurses in a Saudi tertiary hospital use digital communication to coordinate shift swaps, and how do they navigate reciprocity, professional accountability, and access to scheduling flexibility?

## 2. Materials and Methods

### 2.1. Study Design

This qualitative descriptive study used individual semi-structured interviews and digital journey-mapping prompts to examine nurses’ accounts of requesting, negotiating, reviewing, and approving shift swaps. Qualitative description was selected because the study sought a practice-near account of the participants’ experiences without attempting to generate a formal theory [32].

Although focused ethnography can be useful for examining bounded healthcare practices, it requires a defensible ethnographic orientation and appropriate engagement with the setting [33]. The present study relied primarily on interviews rather than sustained ethnographic observation and was therefore classified as qualitative descriptive research.

Data were interpreted using reflexive thematic analysis [34,35]. Because reflexive thematic analysis rests on qualitative values that generic reporting checklists do not always accommodate, the manuscript was additionally appraised against the Reflexive Thematic Analysis Reporting Guidelines (RTARG), which Braun and Clarke developed as a methodologically congruent alternative to COREQ and SRQR [36].

That appraisal shaped several features of this report. Themes are presented as patterns of shared meaning organized around a central concept rather than as topic summaries. Coding is described as an interpretive process rather than an accuracy-seeking one, and no inter-coder reliability is claimed or reported (Section 2.8). The analytic positioning of the team, including the concepts that oriented interpretation, is stated explicitly (Section 2.8 and Section 2.9). No claim of saturation, prolonged engagement, or member checking is made (Section 2.10). SRQR and COREQ were also completed [37,38], since both remain conventional for qualitative reporting in nursing journals, and the COREQ checklist is provided in Supplementary File S2. Where the checklists and the RTARG diverge, the RTARG was followed and the divergence is stated rather than resolved silently; a short RTARG-informed reflexive statement.

### 2.2. Setting and Organizational Context

The study was conducted in one tertiary hospital in the Riyadh region of Saudi Arabia. Participating clinical areas included medical–surgical wards, intensive care units, emergency departments, and specialty units, including oncology and high-dependency care.

The hospital used an electronic rostering system, formal procedures for schedule changes, staffing-escalation pathways, and unit-level managerial oversight. Participants also described using private messages, unit messaging groups, and internal communication channels to identify and negotiate potential swaps before formal approval. These informal channels did not replace the formal rostering system; rather, they allowed nurses to identify a potential replacement before submitting the proposed change for managerial review and roster updating.

### 2.3. Eligibility Criteria

Registered nurses were eligible if they: (1) worked in an inpatient or other shift-based clinical area; (2) had requested, negotiated, coordinated, reviewed, or approved a shift swap during the preceding 6–12 months; (3) had worked in the organization for at least six months; (4) could participate in Arabic or English; and (5) provided written informed consent.

Nurses working exclusively in non-shift administrative roles and temporary staff with less than six months of organizational experience were excluded.

### 2.4. Sampling and Recruitment

Purposive maximum-variation sampling was used to recruit participants with direct experience of shift swapping. Sampling sought variation in clinical area, professional role, nursing experience, and shift pattern.

Recruitment was facilitated through unit announcements, liaison with nursing administration, and participant referral. These approaches were selected to reach nurses in different clinical roles while recognizing the practical challenges of recruiting staff working rotating shifts [39]. Of the 24 participants interviewed, 10 were recruited following unit announcements, 8 through liaison with nursing administration, and 6 through referral by an already participating colleague.

Thirty potential participants were approached. Six declined because of competing work or scheduling commitments. Twenty-four participants provided informed consent and completed interviews, representing a participation rate of 80%. All 24 completed interviews were included in the analysis.

Sample adequacy was considered in relation to the focused research question, specificity and diversity of the sample, quality of interview dialogue, and depth and variation of the accounts. Recruitment concluded when the dataset provided sufficiently rich and varied information to address the research question across the included roles and clinical areas. This assessment was informed by the concept of information power rather than by an assumption that all possible experiences had been exhausted [40].

### 2.5. Data Collection

Data were collected from 1 September to 15 November 2025. Individual semi-structured interviews were conducted in Arabic or English according to participant preference. Fourteen interviews were conducted in Arabic and ten in English. Seventeen interviews were conducted online through a secure, institutionally approved platform, and seven were conducted in private rooms at the participating hospital.

All interviews were conducted by a male researcher who held a PhD in Nursing and had more than 10 years of professional and research experience. The interviewer had no prior personal, clinical, managerial, supervisory, or other professional relationship with the participants. Before each interview, participants were informed about the interviewer’s professional background and the study purpose.

Interviews lasted approximately 45–90 min and were audio-recorded with the participants’ consent. Field notes were completed after each interview to document contextual observations, points of emphasis, and reflexive considerations.

#### Digital Journey Mapping

Digital journey mapping was used as a structured interview technique rather than as a separate data-collection instrument or distinct data source. After the participants described their general experiences of shift swapping, the interviewer asked them to reconstruct one specific recent swap from beginning to end. The prompts included: “Take me through the last swap you were involved in, starting from the point at which you knew you needed one”; “Who did you contact first, and through which channel?”; “What happened next, and roughly how long did that take?”; “At what point did a charge nurse or manager become involved?”; and “When and how did the change appear in the roster?” When a participant moved between two non-adjacent steps, the interviewer asked what had occurred in between.

Participants were not asked to draw, sketch, or submit a diagram, and no participant-generated artifact was collected. Screenshots and identifiable digital messages were also not collected. Instead, the interviewer recorded the reported sequence during and immediately after each interview using a fixed five-field entry in the field-note template: trigger; first contact and channel; negotiation and response; managerial review; and roster documentation. Reported timings, deviations from the usual sequence, and points at which the process stalled were recorded within the same entry. These sequence records remained linked to the corresponding interview and were analyzed as part of the interview corpus. They were not treated as a separate dataset and did not generate themes independently of the interview material.

**Figure 1:**
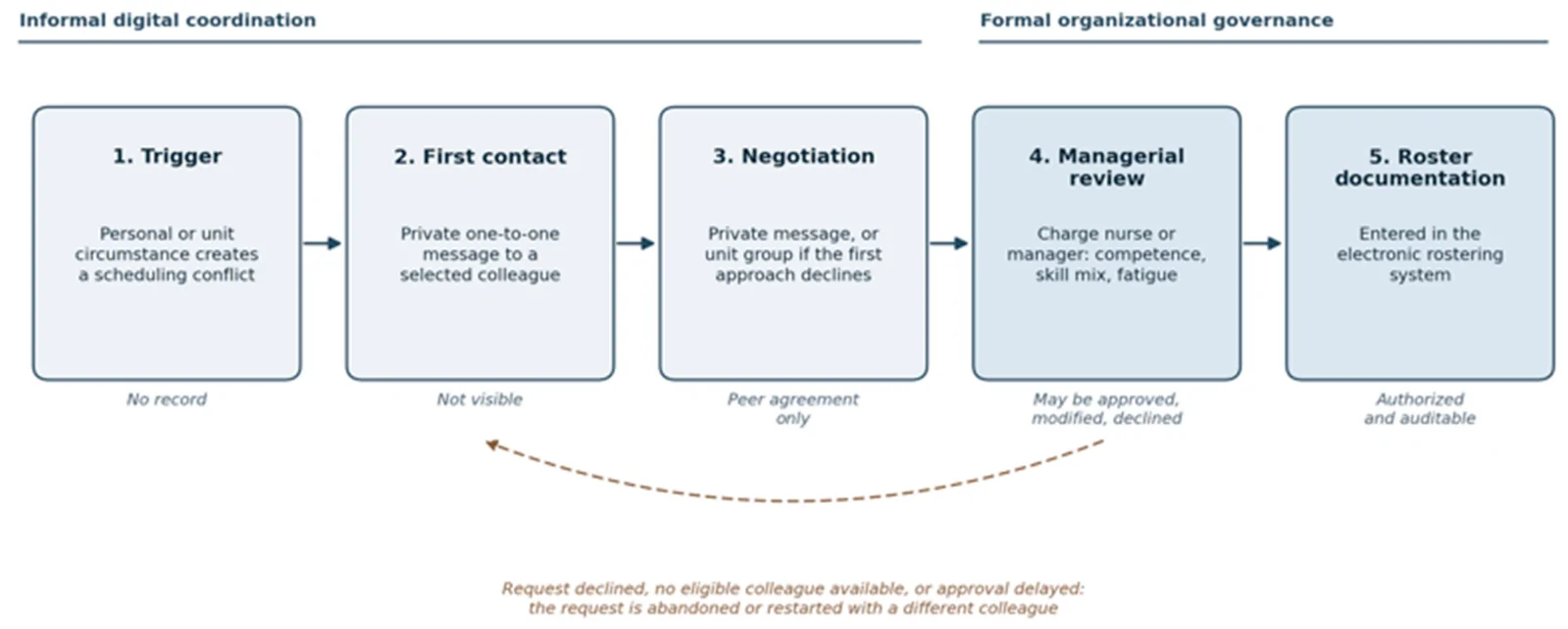
Composite pathway of a digital shift-swap request, reconstructed from participants’ journey-mapping accounts. Solid arrows indicate the sequence described by most participants; the dashed return indicates the point at which a request was commonly abandoned or restarted. The figure aggregates accounts across participants and does not depict any single exchange.

The mapping served two analytic purposes. During familiarization and coding, it made the temporal ordering of each account explicit, supporting analysis of where negotiation, refusal, delay, and authorization occurred in the reported process rather than where participants assumed they occurred. During theme development, sequences were compared across participants to identify the recurring pathway and its common variations, which are summarized in Figure 1. Because the sequences were participant reconstructions rather than direct observations, they describe how participants accounted for the process rather than providing an independently verified record of events (Section 6).

### 2.6. Interview Guide

The semi-structured interview guide was developed for this study, reviewed by researchers with qualitative expertise, and piloted with two nurses who were not included in the final analysis. Piloting was used to assess and refine the clarity, neutrality, relevance, and sequence of the questions. Neutral probes invited participants to provide examples, explain sequences of events, and describe unsuccessful or contrasting experiences. The guide did not assume that shift swapping demonstrated solidarity or produced beneficial outcomes. The complete interview guide is provided in Supplementary File S1.

### 2.7. Transcription, Translation, and Data Management

Audio recordings were transcribed verbatim in the language in which each interview was conducted. Transcripts were checked against the recordings and de-identified before analysis. Names and potentially identifying references to colleagues, units, or specific incidents were removed or generalized. Participants were assigned identifiers from N01 to N24.

The 14 Arabic-language interviews were transcribed in Arabic and translated into English where required for team analysis and reporting. Translation was carried out by two bilingual members of the research team, both native Arabic speakers with postgraduate nursing qualifications taught in English and prior experience translating qualitative healthcare interview data.

Translation followed a forward-translation and independent-review procedure rather than formal back-translation. One translator produced the English version, and the second independently compared the translation line by line with the Arabic transcript. Disagreements were discussed with reference to the original transcript, and where meaning remained unclear, to the audio recording. The criterion applied was conceptual equivalence rather than literal correspondence. Idiomatic Arabic expressions were therefore rendered using the closest English formulation that preserved the participant’s intended meaning, and such translation decisions were documented in the translation log.

Because independent back-translation was not performed, translated quotations are presented as conceptually equivalent English renderings rather than as verbatim English speech. This distinction and its implications for interpretation are acknowledged in Section 3.2 and Section 6.

Audio recordings, transcripts, field notes, translation records, and analytic materials were stored on password-protected, access-restricted institutional or university systems. Access was limited to authorized research-team members.

All reported quotations were verified against the original transcripts. Quotations from English-language interviews reproduce the participants’ words, with only minor punctuation changes or the removal of repeated filler words where required for readability. Quotations translated from Arabic represent conceptually equivalent English translations. These editorial changes did not alter the substance or intended meaning of the participants’ accounts.

### 2.8. Data Analysis

Data were managed using NVivo version 14 (Lumivero, Denver, CO, USA) as an organizational tool and analyzed using reflexive thematic analysis [35]. Examples of reflexive thematic analysis in healthcare research also informed attention to context, researcher interpretation, and alternative explanations [34].

Analysis involved: (1) familiarization through repeated reading of the transcripts and field notes; (2) inductive coding of relevant data segments; (3) examination of relationships among codes; (4) development of candidate themes; (5) review of themes against the coded material and complete dataset; (6) refinement and naming of themes; and (7) production of an interpretive account supported by participant quotations.

Coding was conducted at a predominantly semantic level and stayed close to the participants’ accounts. Codes addressed request initiation, selection of colleagues, communication sequence, responses and refusals, managerial approval, competence, skill mix, documentation, reciprocal expectations, and perceived inequality. No coding frame was fixed before the transcripts were read, and codes were not allocated to predetermined categories.

The analysis was not purely inductive, and describing it as such would misrepresent it. Reciprocity and professional accountability were named in the research question and developed in the Introduction, so the team approached the dataset already oriented toward them; these two concepts are best understood as orienting constructs present from the outset. Hierarchy, peer support, workplace social capital, and solidarity entered later, during theme development, as sensitizing concepts used to interpret patterns that had already been generated from the coded material. The analysis is therefore more accurately characterized as inductively driven at the level of coding and theoretically informed at the level of interpretation.

Two safeguards were applied against reading the orienting concepts into the data. First, the interview guide was written so as not to presuppose them: it asked what happened, who was contacted, in what order, and with what outcome, and it invited accounts of refused, unsuccessful, and resented swaps (Supplementary File S1). Second, during theme review, the team deliberately searched for material that did not fit a reciprocity reading, such as assistance given without expectation of return, requests declined without relational consequence, and accounts framed in purely procedural terms. Solidarity was retained only as a qualified interpretation where participants’ accounts supported reciprocal assistance and collective coping, and Section 3.2.4 reports the conditions under which that reading did not hold.

Three researchers participated in the analysis. They reviewed the interview material and discussed codes, candidate themes, contradictory accounts, and alternative interpretations. These discussions were intended to deepen reflexive engagement rather than calculate inter-coder reliability or establish one objectively correct coding structure. Differences in interpretation were examined to identify assumptions, consider alternative readings, and refine the themes.

Reflexive memos documented analytic decisions, theme development, alternative explanations, and distinctions between the participants’ accounts and researchers’ interpretations. The final themes were reviewed against the complete dataset for internal coherence, conceptual distinction, evidential support, and relevance to the research question.

### 2.9. Researcher Reflexivity

The research team considered how professional backgrounds, institutional affiliations, familiarity with Saudi nursing contexts, and assumptions about scheduling flexibility could affect recruitment, interviewing, analysis, and reporting.

The interviewer maintained reflexive notes concerning how his nursing background, gender, questions, and responses might have shaped the interview interactions. The analytic team specifically questioned assumptions that shift swapping was necessarily beneficial, demonstrated solidarity, or improved patient safety.

Alternative interpretations involving obligation, exclusion, hierarchy, professional identity, and the transfer of scheduling work from the organization to individual nurses were considered during theme development. Professional identity and workplace position can shape how nurses interpret their responsibilities and interactions [41], while hierarchy may affect willingness to speak, refuse, or raise concerns [21,22].

### 2.10. Trustworthiness

Trustworthiness was addressed through credibility, dependability, confirmability, and transferability [42].

Credibility was supported through maximum-variation sampling, inclusion of frontline and leadership perspectives, detailed interviewing, repeated engagement with the transcripts, examination of contrasting and contradictory accounts, and use of quotations to demonstrate the evidential basis of interpretations.

Dependability was supported by retaining the interview guide, recruitment records, field notes, coding records, reflexive memos, translation documentation, and theme-development records. Confirmability was supported by distinguishing the participants’ accounts from the researchers’ interpretations and documenting alternative explanations.

Transferability was supported through description of the setting, sample, professional roles, digital coordination process, and formal approval requirements. The findings are intended to support reasoned comparison with similar settings rather than statistical generalization.

Claims of prolonged engagement, saturation, or participant validation were not made because these procedures were not sufficiently established in the study documentation.

### 2.11. Ethical Considerations

Ethical approval was obtained from the Institutional Review Board of King Saud University (Ref. No. KSU-HE-25-906) on 5 August 2025. Data collection commenced on 1 September 2025 after ethical approval had been granted, and concluded on 15 November 2025.

Written informed consent was obtained before each interview. Consent included permission to audio-record the interviews and publish de-identified quotations. Participants were informed about the study purpose, voluntary participation, confidentiality, data management, and their right to withdraw without adverse consequences.

Participants were instructed not to disclose identifiable patient, colleague, or message content. Transcripts and quotations were de-identified, and unnecessary contextual descriptors were omitted to reduce the risk of deductive identification.

## 3. Results

### 3.1. Recruitment and Participant Characteristics

Thirty potential participants were approached. Six declined because of competing work or scheduling commitments. Twenty-four registered nurses provided informed consent, completed an interview, and were included in the analysis.

The sample included 16 staff nurses, five charge nurses, and three nurse managers or clinical coordinators. Participants were 23–49 years old and had 2–24 years of nursing experience. Fourteen participants were women and ten were men.

Participants represented medical–surgical, intensive care, emergency, and specialty units. Sixteen reported participating in at least one shift swap per month, whereas eight described occasional or situational involvement. All participants reported using the electronic rostering system alongside at least one additional digital communication channel. Full participant characteristics are presented in Table 1.

### 3.2. Thematic Findings

Four interrelated themes were developed: (1) digital pathways of negotiation; (2) reciprocity as social currency; (3) managing risk and responsibility; and (4) collective coping and unequal flexibility.

Together, the themes show that informal communication enabled nurses to explore potential swaps, whereas managerial approval and formal roster documentation determined whether an arrangement was authorized. Reciprocal support could increase flexibility but was not equally accessible or free from obligation.

Two reporting conventions apply throughout this section. First, quotations from interviews conducted in Arabic are conceptually equivalent English translations produced through the procedure described in Section 2.7, not verbatim English speech; quotations from interviews conducted in English reproduce the participants’ words with minor punctuation adjustment and the removal of repeated filler only. Every quotation carries its participant identifier (N01–N24).

Second, to indicate how widely a pattern was expressed, the quantifiers most (18 or more of the 24 participants), many (12–17), some (6–11), and a few (2–5) are used consistently, based on the number of participants whose coded material contributed to each subtheme. The number of contributing participants is reported for each subtheme in Table 2. These counts describe how accounts were distributed within this sample and are reported for transparency; they are not a measure of the importance or strength of a theme, and reflexive thematic analysis does not treat them as one.

#### 3.2.1. Digital Pathways of Negotiation

Participants described shift swapping as a multistep process rather than a single electronic transaction. Requests moved between private messages, group chats, conversations with managers, and the formal rostering system. The sequence depended on the urgency and sensitivity of the request and established unit practices.

##### Private Contact and Group Visibility

Most participants preferred to contact a colleague privately before posting a request in a group. This approach allowed the recipient to decline without embarrassment or perceived public pressure:

*“You don’t usually put it in the group first. You message the person privately and ask if they’re free. If they say yes, then you put it in the group.”*

(N07)

After reaching an agreement privately, some participants shared the proposed arrangement in the unit group so that other team members were aware of it:

*“If we agree privately, then we write it in the group so everyone knows. It’s more transparent that way.”*

(N12)

This sequence was not universal. When a nurse became suddenly ill or another urgent situation arose, some participants posted directly in the group to identify an available colleague quickly. Digital coordination therefore involved balancing privacy and relational sensitivity against the need for a rapid response.

##### Approval and Documentation

Most participants clearly distinguished between reaching an agreement with a colleague and obtaining formal approval. A proposed swap was not considered final until it had been approved by the appropriate manager and entered into the official roster:

*“Even if we agree between ourselves, it doesn’t really count until it’s approved and put in the system.”*

(N03)

Charge nurses and managers reviewed whether the replacement nurse had the required competence and whether the proposed arrangement would preserve adequate staffing:

*“We check if the replacement nurse has the right competencies. It’s not just about helping someone; we have to make sure the coverage is safe.”*

(N18)

Most participants were concerned about leaving an arrangement undocumented because they understood the official roster, rather than an informal message, to determine who was accountable for the shift:

*“If something happens and the system still has your name, you’re responsible. So we always make sure it’s updated.”*

(N21)

Formal procedures were therefore experienced as both protective and restrictive. They clarified responsibility and supported oversight but could delay urgent arrangements, particularly outside the usual managerial working hours. The sequence of actions and decision points involved in a digital shift-swap request is illustrated in Table 3.

#### 3.2.2. Reciprocity as Social Currency

Participants’ willingness to assist was often shaped by their previous relationships with colleagues rather than availability alone. They remembered who had helped them previously, and this relational history influenced their willingness to provide assistance in return.

##### Remembered Assistance

Many participants described an implicit expectation that help would eventually be reciprocated:

*“If someone covered my night shift last month, of course I’ll help them now. That’s just understood.”*

(N09)

Reciprocity facilitated mutual assistance but could also create pressure. Some participants accepted inconvenient swaps because they anticipated needing similar assistance in the future:

*“Sometimes you say yes even when it’s difficult, because you know that one day you might need their help.”*

(N15)

Some participants also described small groups of colleagues who regularly exchanged shifts with one another. These networks increased flexibility for their members but could make assistance more difficult to obtain for nurses outside them. Reciprocity was therefore experienced as both supportive and obligating.

##### Selective Asking and Timing

Most participants considered carefully whom to approach and when to make a request. Their decisions reflected a colleague’s fatigue, previous assistance, personal circumstances, competence, and perceived likelihood of accepting the swap:

*“You don’t ask someone who has just finished three night shifts. You think first—who is more likely to say yes?”*

(N04)

Requests made well in advance were generally considered more acceptable, whereas last-minute requests were expected to have a compelling reason:

*“If you ask at the last minute, it should be because something serious happened. Otherwise, people feel it’s not fair.”*

(N19)

Many participants provided a brief explanation to establish why the request was necessary while avoiding excessive disclosure of personal information. Shift-swap coordination therefore involved assessing the situation, selecting an appropriate colleague, and negotiating how much personal information to disclose, rather than simply communicating a scheduling request.

#### 3.2.3. Managing Risk and Responsibility

Participants viewed flexibility as professionally bounded. Helping a colleague was valued, but it could not override requirements concerning competence, staffing, workload, fatigue, approval, and documentation.

##### Protecting Staffing and Competence

Most participants emphasized that nurses were not necessarily interchangeable, particularly in higher-acuity settings:

*“It’s not just about filling the slot. You have to think, can this nurse actually manage these patients?”*

(N02)

Proposed swaps could be declined when a nurse’s experience or competence did not correspond to the requirements of the unit or shift:

*“We help each other, but if the unit becomes unsafe, then it’s not right. Patient safety comes first.”*

(N11)

Some participants also described declining requests when they considered themselves too fatigued to work appropriately:

*“If I’m too tired, I’ll say no. It’s not fair to the patients.”*

(N17)

These accounts demonstrate how participants reasoned about safe staffing and professional responsibility. They do not establish that shift swapping itself affected patient outcomes.

##### Accountability and Policy

Most participants were particularly concerned when a peer agreement had not been formally recorded. The account quoted in Section 3.2.1 (N21) illustrates the reasoning: participants understood the roster, rather than a message thread, to determine who carried responsibility for a shift, and the same concern was raised independently by participants across all four clinical areas.

Some participants also noted that the charge nurse needed to be informed, and that failure to report a swap could be interpreted as an attempt to bypass established procedures:

*“You have to tell the charge nurse. If they find out later, it looks like you were trying to hide it.”*

(N05)

Some junior participants appeared more concerned that requesting a swap could make them seem unreliable or suggest that they were attempting to circumvent the usual hierarchy. More experienced nurses described greater confidence in navigating approval procedures, although they remained subject to the same formal requirements.

#### 3.2.4. Collective Coping and Unequal Flexibility

Shift swaps helped participants manage unexpected events and competing responsibilities, including personal emergencies, caregiving, examinations, religious commitments, and periods of increased unit demand. However, access to this flexibility was not experienced equally.

##### Responding to Pressure

Many participants described how colleagues responded collectively when an unexpected absence affected an already busy unit:

*“In an emergency, you never know what’s going to happen. If someone can’t come, we try to solve it together quickly.”*

(N06)

Mutual assistance was especially valued during periods of high workload and fatigue:

*“When the unit is busy and everyone is exhausted, helping each other is what keeps us going.”*

(N14)

Some charge nurses considered a reasonable degree of flexibility important for staff morale:

*“If we refuse every request, people will feel trapped. Then morale goes down.”*

(N22)

Some participants also described an increased need for flexibility during Ramadan and examination periods. These accounts suggest that shift swapping could contribute to collective coping, and under some circumstances, be experienced as professional solidarity. They do not demonstrate that shift swapping prevented burnout, absence, or staffing shortages.

##### Relational Inequalities

Many participants described differences in how readily nurses obtained assistance. Successful requests could depend on seniority, familiarity, perceived competence, trust, and inclusion in informal networks:

*“Some people always get help quickly. Others have a hard time finding someone.”*

(N10)

Being regarded as reliable could make assistance easier to obtain:

*“If you’re experienced and people trust you, it’s easier. They know you’ll return the favor.”*

(N18)

Some participants perceived that language or cultural differences affected inclusion in informal networks, although they did not consistently describe this as deliberate exclusion. Their principal concern was that reliance on these networks could produce unequal access to flexibility:

*“We say we are one team, but sometimes flexibility depends on who you know.”*

(N03)

The findings therefore do not indicate that solidarity was experienced uniformly. Instead, solidarity was conditional: collegial support helped nurses manage difficult circumstances, but access to that support was shaped by interpersonal relationships, hierarchy, and position within informal networks.

## 4. Discussion

### 4.1. Principal Findings

This study examined how nurses in one Saudi tertiary hospital used digital communication to coordinate shift swaps and navigated reciprocity, accountability, and access to flexibility. The central finding has three connected parts. Shift swapping operated as a hybrid coordination practice: the substantive work of finding a replacement was carried out informally and relationally through peer messaging, the authority to change the schedule rested entirely with managerial approval and the electronic roster, and the interval between the two was bridged by the nurses themselves. Because that bridging work drew on relational resources, access to flexibility tracked the distribution of those resources rather than the distribution of need.

### 4.2. Interpretation in Relation to Previous Research

Participants moved between private messages, group communication, managerial contact, and formal rostering. Private communication served a relational function by reducing public pressure and preserving the recipient’s ability to decline; group communication provided visibility; the formal roster established accountability.

This extends administrative accounts of nurse rostering [3] by making visible the interpersonal work that precedes a formal schedule change, and it is consistent with evidence that coordination is negotiated among professionals across organizational boundaries rather than achieved by systems alone [4,31]. Healthcare communication technologies are embedded in organizational practice rather than functioning as neutral tools [5,7,8]. Research on secure messaging and smartphone communication indicates that such tools accelerate communication while introducing concerns about interruption, privacy, information governance, and documentation [10,11], and survey evidence shows that professional use persists at near-universal levels even where organizational guidance discourages it [12]. Informal communication is therefore best understood as a preparatory and relational layer whose value depends on the governance of the wider digital environment [6], not as a substitute for the scheduling system.

Reciprocity was central to the negotiation. Previous assistance influenced whom participants approached and whether requests were accepted, which is consistent with workplace social-capital accounts in which trust and networks provide access to relational resources [16]. It was not uniformly beneficial. The mechanism that made assistance available could also pressure nurses into accepting inconvenient requests in order to preserve future access to support, so informal networks functioned simultaneously as a resource and as a boundary. Scholarship on relational power similarly suggests that reciprocal interaction can generate both agency and constraint, although the nurse–patient context differs substantially from peer scheduling relationships [18].

The findings provide qualified evidence of professional solidarity where nurses responded collectively to personal or unit-level pressures [17]. Solidarity is nonetheless better treated as a secondary interpretive concept here. Participants’ accounts more directly demonstrate reciprocal peer coordination, collective coping, and unequal access to flexibility.

Setting these findings alongside rostering research clarifies what is at stake. Barker and colleagues found that roster decisions emerge from conflict between the priorities of nursing staff, unit managers, and senior leaders, and that collaborative and compromising responses contained that conflict whereas rigid or competing responses escalated it [30]. The present study identifies a second site at which the same conflict is worked out, one that roster-level research does not usually observe: the peer-to-peer negotiation preceding the manager’s decision. Fairness in that space is not produced by an allocation rule. It is produced by who is asked, who feels able to refuse, and whose request is treated as reasonable. Research on informal workplace flexibility points the same way, finding that access to informal adjustment is mediated by line managers and co-workers rather than granted by entitlement, and that employees without those relationships are correspondingly disadvantaged [43]. Where participants here described flexibility as depending on personal connection, they were describing that mediation operating among peers.

Informal shift exchanges also fill gaps between formal workplace arrangements and employees’ changing personal circumstances [23]. That function warrants closer attention than it usually receives, because it involves a transfer of work. When a roster cannot accommodate a nurse’s caregiving obligation, illness, examination, or religious commitment, the shortfall does not disappear. It is displaced onto the nurse, who must then locate a willing colleague, judge whether that colleague is competent for the shift, manage the reciprocal obligation the request creates, and secure authorization. Each of those tasks is unpaid, undocumented, and invisible to workforce planning, and the capacity to perform them is unevenly distributed across the workforce. Comparable dynamics have been described in the wider literature on informal flexibility, where employees’ ability to adjust their work depends on managerial discretion and colleague goodwill rather than on entitlement [43]. A well-functioning swap culture may therefore indicate that nurses are absorbing scheduling shortfalls on the organization’s behalf, and it should be read alongside evidence on staffing adequacy and workload rather than as a substitute for it [24,29].

Participants did not regard peer agreement as sufficient. They described competence, skill mix, fatigue, workload, approval, and documentation as boundaries on acceptable swaps, so informal flexibility was integrated with, rather than opposed to, professional accountability. Managerial review remained important because a mutually convenient exchange could still produce an inappropriate distribution of competence, experience, or workload; collaboration and management support may facilitate appropriate decisions [19,20], whereas hierarchical cultures can make staff reluctant to challenge unsafe or unfair arrangements [21,22]. Documentation was more than an administrative step: participants viewed roster updating as establishing responsibility and protecting them from ambiguity, and delayed approval during nights and weekends created a gap between peer agreement and formal authorization.

These findings should not be read as evidence that shift swapping improved or harmed patient safety. Research has shown relationships among patient acuity, staffing, missed care, and outcomes [29], but the present study measured none of these. Participants reported considering safety-related factors when assessing proposed arrangements; whether those considerations produced safe outcomes was not assessed.

Seniority, trust, social integration, and perceived competence influenced access to successful swaps. Junior nurses could be reluctant to ask for fear of appearing unreliable, whereas established nurses possessed greater relational resources; digital communication increased the speed of coordination without removing workplace hierarchy. Professional identity develops through interactions, organizational expectations, and transitions into practice [41], and nurses newer to a unit may have fewer relational resources and less confidence in negotiating exceptions. Hierarchical unit culture and perceived futility of speaking up may compound this [21]. A process resting predominantly on personal networks can therefore produce unequal opportunity even where no participant intends to exclude anyone. Whether transparent organizational procedures would reduce that unevenness was not examined in this study.

### 4.3. Interpretation Within the Saudi Context

The findings should be interpreted within one tertiary hospital rather than generalized to Saudi nursing as a whole. The setting combined electronic rostering, formal hierarchical approval, shift-based nursing, workforce diversity, and expanding healthcare digitalization under national health-system transformation [25,26].

Research involving Saudi nurses has identified interactions among professional identity, cultural expectations, workplace relationships, and retention [27,28]. References to Ramadan, language differences, and professional hierarchy in the present study provide context-specific insights and should not be used to infer fixed cultural attributes or characterize all Saudi nurses as collectivist.

The principal contextual contribution of this study is organizational: nurses negotiated flexibility within a digitally developing, professionally diverse, and formally governed hospital environment.

### 4.4. Implications for Scheduling Technology

Digital scheduling systems could potentially support secure requests, competence checks, managerial review, transparent approval status, and auditable documentation. Research on AI-supported scheduling has begun to examine nurses’ concerns about fairness, transparency, and work–life balance [15]. Such systems should not be assumed to be neutral or automatically beneficial.

Technology acceptance, usability, governance, and organizational readiness remain important [5,8]. Digital change also unfolds over time and may alter the pace, location, and boundaries of work [9]. Participants’ accounts suggest that nurses’ involvement in system design, and the evaluation of effects on fairness, workload, communication burden, privacy, and staffing, would be pertinent to any such implementation. No implementation of this kind was observed or assessed here.

Two limits on this section should be explicit. First, this study evaluated no scheduling technology, AI-based or otherwise: no system was trialed, observed, or assessed, and participants were not asked about any specific product. Second, the dimensions named above, namely fairness, transparency, privacy, workload, communication burden, and equity of access, are proposed design considerations derived from what the participants said about their existing arrangements. They are not intervention recommendations, and they carry no claim that a system built around them would improve any workforce or patient outcome. Whether these are the right dimensions, and whether attending to them changes anything, are empirical questions that this design cannot answer.

## 5. Implications

The following implications are inferred from participants’ accounts in one hospital and were not tested against measured outcomes. Because the design was qualitative and the setting was a single hospital, they are described as considerations for local discussion and as propositions for future evaluation rather than as recommendations for practice.

### 5.1. Nursing Practice

Clear guidance on shift-swap procedures, documentation, competence, fatigue, and professional boundaries may support safer coordination. Participants placed weight on being able to decline a request, whether on grounds of competence, fatigue, or personal circumstance, without retaliation or perceived loss of future flexibility, and on treating a peer agreement as provisional until approval and roster updating were completed. The literature on clinical messaging raises a related question about identifiable patient information and unnecessary personal detail in informal channels [10,11,14]. These points describe what mattered to nurses in one hospital rather than practices shown here to improve coordination or safety.

### 5.2. Nursing Management

Several areas that local managers might discuss are apparent in the participants’ accounts: transparent criteria for competence, skill mix, workload, fatigue, and approval responsibilities; equitable distribution of unpopular shifts; documentation; and protection against coercive reciprocal expectations. Participants also described occasions on which no appropriate swap could be arranged, which points to escalation routes as a further area for local attention. Possible differences in access to flexibility among junior, newly employed, or less socially integrated nurses may merit local attention, although this study did not measure them.

### 5.3. Digital Workplace Systems

Where feasible, electronic rostering systems could support secure swap proposal, approval, and documentation. Relevant features may include clear approval status, audit trails, privacy protection, notification controls, eligibility criteria, and appropriate competence and fatigue safeguards. Whether such features would help was not tested here, and their development would call for co-design with nurses and evaluation before wider implementation.

### 5.4. Organizational Policy

The distinction between informal peer communication and authorized roster changes was salient in the participants’ accounts, as were questions about acceptable communication channels, approval responsibilities, data protection, fatigue and skill-mix requirements, and procedures for urgent requests. These are candidate elements for local policy review rather than provisions this study has shown to be effective. The findings also give no indication that peer support can substitute for organizational responsibility regarding adequate staffing and equitable scheduling.

## 6. Strengths and Limitations

This study included staff nurses, charge nurses, and nurse managers from several acute-care areas, capturing both peer negotiation and managerial oversight. Maximum-variation sampling included diverse roles, experience levels, and clinical contexts. Journey mapping generated sequential accounts of shift-swap coordination, while attention to refusal, obligation, hierarchy, and unequal access avoided portraying swaps as uniformly beneficial.

Several limitations should be noted. First, the single tertiary hospital setting may limit transferability to other regions, sectors, primary-care settings, or staffing models. Second, recruitment through unit announcements, nursing administration, and participant referral may have introduced self-selection and network effects. Third, the interviewer’s nursing background, gender, and professional status may have influenced disclosure despite reflexive practices. Fourth, retrospective self-reports were not independently verified against messages, roster changes, or approval records. Fifth, Arabic-to-English translation may have affected nuance despite bilingual checking; translated quotations are conceptually equivalent renderings. Sixth, online and in-person interviews may have differed in interpersonal dynamics and access to non-verbal cues. Seventh, journey mapping reflected participant reconstructions rather than observed records, and Table 3 is a composite illustration. Eighth, participant counts describe the distribution of coded material within the sample, not theme importance or prevalence. Finally, the study did not assess patient safety, burnout, absenteeism, retention, missed care, or organizational resilience, which require separate evaluation.

## 7. Future Research

Four directions follow from these limitations. First, multicenter studies across regions, sectors, hospital sizes, and staffing models should examine whether the pathway identified here extends beyond this organization. Second, studies linking participants’ accounts with ethically and legally accessible records, such as de-identified messages, roster changes, and approval timestamps, could compare reported and documented processes. Third, digital shift-swap functions should be co-designed with nurses and evaluated in pilot studies against predefined outcomes. Fourth, longitudinal studies should examine whether transparent procedures improve perceived fairness, whether reliance on informal networks contributes to unequal access to flexibility, and whether these patterns are associated with burnout, absence, missed care, or intention to leave.

A further priority is the distribution of flexibility. Differences related to seniority, tenure, nationality, and language were evident in the participants’ accounts but could not be quantified. Future studies combining roster data with measures of procedural fairness could assess these differences more directly.

## 8. Conclusions

Nurses in the single tertiary hospital studied here described digital shift-swap coordination as a relational and professionally bounded practice operating alongside formal rostering. Digital communication facilitated negotiation, while managerial approval and roster documentation established accountability.

Reciprocal peer support helped nurses respond to personal and workplace pressures but could also create obligation and unequal access to flexibility. Seniority, trust, familiarity, and inclusion in informal networks affected how readily nurses obtained assistance.

Transparent procedures connecting peer communication with formal approval may preserve responsiveness while supporting fairness, documentation, professional boundaries, and appropriate staffing. This is a proposition drawn from one purposively recruited sample in one hospital rather than a finding about Saudi hospitals in general, and it requires testing. Whether digital solutions developed together with nurses would alter equity, privacy, workload, and communication expectations remains an open question, and one that would need evaluation before wider implementation.

## Figures and Tables

**Table 1:** Participant characteristics (*n* = 24).

Characteristic	Category	*n*	%
Age, years	23–30	8	33.3
	31–35	3	12.5
	36–40	7	29.2
	>40	6	25.0
Gender	Women	14	58.3
	Men	10	41.7
Highest qualification	Bachelor’s degree	19	79.2
	Master’s degree	5	20.8
Primary clinical area	Medical–surgical	8	33.3
	Intensive care	6	25.0
	Emergency	5	20.8
	Specialty/HDU/oncology	5	20.8
Nursing experience	2–4 years	5	20.8
	5–10 years	9	37.5
	11–15 years	6	25.0
	>15 years	4	16.7
Professional role	Staff nurse	16	66.7
	Charge nurse	5	20.8
	Nurse manager/coordinator	3	12.5
Shift-swap frequency	At least monthly	16	66.7
	Situational/occasional	8	33.3
Interview language	Arabic	14	58.3
	English	10	41.7
Interview mode	Online	17	70.8
	In person	7	29.2

HDU: high-dependency unit.

**Table 2:** Themes, subthemes, and analytical descriptions.

Theme	Subtheme	Analytical Description
Digital pathways of negotiation	Private contact and group visibility [*n* = 21]	Nurses moved between private and group communication according to sensitivity, urgency, and unit norms.
	Approval and documentation [*n* = 22]	Peer agreement required managerial review and formal roster updating.
Reciprocity as social currency	Remembered assistance [*n* = 17]	Previous assistance influenced current willingness to accept requests.
	Selective asking and timing [*n* = 19]	Nurses assessed relationships, fatigue, competence, and urgency before requesting help.
Managing risk and responsibility	Protecting staffing and competence [*n* = 20]	Proposed swaps were assessed in relation to skill mix, fatigue, workload, and staffing requirements.
	Accountability and policy [*n* = 18]	Formal approval and documentation were treated as necessary safeguards.
Collective coping and unequal flexibility	Responding to pressure [*n* = 16]	Shift swaps helped nurses respond to personal emergencies and unit demands.
	Relational inequalities [*n* = 13]	Seniority, trust, familiarity, and social integration affected access to flexibility.

Note: Counts in square brackets indicate the number of participants, out of 24, whose coded material contributed to each subtheme. The counts describe the distribution of coded accounts within the sample and should not be interpreted as measures of theme importance or strength. The quantifiers most (18 or more participants), many (12–17), some (6–11), and a few (2–5) are used consistently throughout the Results section based on these participant-level contributions.

**Table 3:** Illustrative composite pathway of a digital shift-swap request.

Stage	Channel Described	Typical Content of the Exchange	Governance Status at this Stage
1. Trigger	None (personal circumstance)	A nurse identifies a conflict between an assigned shift and a personal obligation, for example, illness, caregiving, an examination, or a religious commitment.	No organizational record exists.
2. First contact	Private one-to-one message	The nurse approaches a selected colleague privately, states the shift and date, gives a brief reason, and asks about availability. Selection reflects prior assistance, perceived competence for the unit, and the colleague’s recent roster.	Not visible to the unit or to management; refusal carries no formal or public consequence.
3. Negotiation	Private message, or unit group if the first approach declines	Agreement is reached on which shifts are exchanged in each direction. Where the private approach fails or the situation is urgent, the request is posted to the unit group so that a wider set of colleagues can respond.	A peer agreement exists but has no organizational standing.
4. Review	Message or in-person contact with the charge nurse or manager	The proposed exchange is submitted for review against competence and scope for the receiving unit, skill mix across the affected shifts, fatigue and consecutive-shift limits, and overall staffing.	The proposal may be approved, modified, or declined. Delays were reported outside the usual managerial hours.
5. Roster entry	Electronic rostering system	The approved exchange is entered into the roster, at which point the named accountable nurse for each shift changes.	The change is authorized and auditable. Until this point, participants regarded the originally rostered nurse as accountable.

The sequence in Table 3 is a composite constructed from the participants’ journey-mapping accounts across clinical areas. It is not a transcript of any single exchange, and no message content was collected in this study (section on Digital Journey Mapping). It is presented because the distinction it makes visible, between stages 3 and 5, was the distinction the participants themselves treated as decisive: the exchange was socially settled at stage 3 and organizationally settled only at stage 5, and the interval between the two is where accountability was described as ambiguous.

## Data Availability

Complete interview transcripts are not publicly available because they contain potentially identifiable information concerning participants, colleagues, and workplace practices. De-identified excerpts may be available from the corresponding author upon reasonable request, subject to ethical, institutional, and data-governance approval.

## Supplementary Materials

The following supporting information can be downloaded at: https://www.mdpi.com/article/10.3390/healthcare14182957/s1, Supplementary File S1: Semi-structured interview guide, including the digital journey-mapping prompts; Supplementary File S2: COREQ 32-item checklist [38].
