# Supplementary material for: Informal Digital Coordination of Nurse Shift Swaps in a Saudi Tertiary Hospital: A Qualitative Descriptive Study
Source: Healthcare (Basel). 2026 Sep 10;14(18):2957. doi: 10.3390/healthcare14182957 (PMC13606776; doi:10.3390/healthcare14182957)
Supplement: Supplementary file 1 [file healthcare-14-02957-s001.zip › healthcare-4537158-supplementary.pdf]

Supplementary File S1: Semi-Structured Interview Guide

Informal Digital Coordination of Nurse Shift Swaps in a Saudi Tertiary Hospital

A. Introduction and Rapport

Participant-facing introduction

Thank you for agreeing to speak with me today. I am a nurse researcher conducting this study to understand how nurses arrange and coordinate shift changes or exchanges with their colleagues and how they communicate during this process.

B. Participant Background

These questions should be used to establish relevant contextual variation without collecting unnecessary identifying information.

B1. Professional background

Can you tell me a little about your current nursing role and your general clinical area?

Probes:

- How long have you worked as a registered nurse?
- How long have you worked in your current clinical area?
- What type of shift pattern do you normally work?

B2. Experience with shift changes

Can you tell me about your experience with arranging or exchanging shifts with colleagues?

Probes:

- How often does this occur in your experience?
- Is this something you usually initiate, respond to, or both?
- Has your experience changed over time?

C. Exploring the Shift-Change Process

C1. A recent experience

Can you walk me through a recent experience of arranging a shift change or exchange, from when the need first arose until the arrangement was finalized?

Probes:

- What happened first?
- Who did you contact?
- Why did you contact that person?
- What happened next?
- How was the arrangement eventually confirmed?
- Was anything different from what you initially expected?

Follow-up prompt:

Can you tell me more about what happened at that point?

C2. How communication takes place

How do you usually communicate with colleagues when discussing a possible shift change or exchange?

Probes:

- What communication methods do you normally use?
- Do you use private messages, group chats, telephone calls, face-to-face conversations, or other methods?
- How do you decide which method to use?
- Does the method change as the request progresses?
- What happens after an arrangement is reached?

C3. Choosing whom to approach

When you need to change or exchange a shift, how do you decide whom to approach?

Probes:

- What factors do you consider?
- Does familiarity with a colleague matter?
- Does the colleague's role or experience matter?
- Does their usual shift pattern matter?
- Can you describe a situation where you approached someone you did not normally ask?

C4. Private communication and group communication

Can you tell me about your experiences of discussing shift changes privately with colleagues?

Probes:

- What makes private communication useful?
- Are there situations in which you prefer to approach someone privately?
- What happens if the request is discussed in a group?
- How does the presence of other colleagues affect the interaction?

Follow-up:

Can you give me an example of a situation where the way the request was communicated mattered?

C5. What makes a shift change possible or difficult

What tends to make a shift change or exchange easier or more difficult to arrange?

Probes:

- Staffing requirements?
- Workload?
- Shift type or timing?
- Colleague availability?
- Clinical experience or competence?
- Existing relationships between colleagues?
- Managerial requirements?
- Formal roster procedures?

Avoid introducing these factors unless relevant; use them only as follow-up prompts when needed.

D. Relationships, Expectations, and Responses

D1. Responding to requests

When another nurse asks you to change or exchange a shift, how do you usually decide whether to agree?

Probes:

- What do you consider before responding?
- Does the reason for the request matter?
- Does your relationship with the colleague matter?

- Have you ever agreed when it was inconvenient for you?
- Have you ever declined a request?

D2. Declining a request

Can you tell me about what happens when someone cannot or does not want to accept a shift-change request?

Probes:

- How is the response usually communicated?
- What happens afterward?
- How do people generally respond to a refusal?
- Have you ever felt uncomfortable declining a request?
- Can you describe a specific example?

D3. Expectations between colleagues

What, if anything, do nurses expect from one another when helping with shift changes?

Probes:

- Do nurses tend to remember previous favors?
- Does helping someone affect future interactions?
- Are there situations where people feel expected to help?
- Can you describe an example?
- Are these expectations openly discussed or understood informally?

E. Formal Rostering and Managerial Involvement

E1. Relationship between informal and formal processes

How does communication between nurses about shift changes relate to the hospital's formal rostering process?

Probes:

- At what point does the formal roster become involved?
- What happens after nurses reach an informal agreement?
- Is managerial or charge-nurse approval required?

- Does the formal process ever differ from what nurses have already agreed?
- Can you describe an example?

E2. Charge nurses and managers

What role do charge nurses or managers play when nurses arrange shift changes?

Probes:

- When are they usually involved?
- What factors influence their decision?
- Have you experienced a request being approved or declined?
- What happens when managerial expectations differ from the nurses' arrangement?

F. Staffing, Workload, Competence, and Accountability

F1. Practical considerations

What kinds of practical considerations need to be taken into account before a shift change can go ahead?

Probes:

- Staffing levels?
- Workload?
- Clinical area?
- Experience or competence?
- Fatigue?
- Consecutive shifts?
- Documentation?
- Managerial approval?

F2. Responsibility and accountability

When nurses arrange a shift change between themselves, how is responsibility understood among the people involved?

Probes:

- Who is responsible for confirming the change?

- What happens if the arrangement is not formally documented?
- Have you experienced any misunderstanding about who was responsible?
- Can you describe an example?

G. Digital Communication and Visibility

G1. Digital communication

What role does digital communication play in arranging shift changes in your workplace?

Probes:

- Which digital channels are commonly used?
- How are they different from face-to-face communication?
- What are the advantages?
- What are the difficulties?

G2. Visibility and privacy

How does it affect the process when a request is visible to several colleagues rather than communicated privately?

Probes:

- Does visibility affect who responds?
- Does it affect whether someone feels comfortable declining?
- Are there situations where you would avoid posting in a group?
- Can you describe an example?

G3. Moving between communication channels

Have you experienced a shift-change discussion moving from one communication method to another?

Probes:

- What prompted the change?
- Did it begin privately and move to a group?
- Did it move from informal communication to the formal rostering system?
- What happened at each stage?

H. Difficulties, Disagreements, and Unexpected Situations

H1. Misunderstandings

Have you experienced a misunderstanding related to a shift change or exchange?

Probes:

- What happened?
- How was the misunderstanding identified?
- How was it resolved?
- What did you learn from the experience?

H2. Disagreement

Can you describe a situation in which nurses disagreed about a proposed shift change?

Probes:

- What was the source of the disagreement?
- Who became involved?
- How was it resolved?
- Did the disagreement affect subsequent interactions?

H3. Situations that did not follow the usual pattern

Can you tell me about a time when arranging a shift change did not happen in the way it normally does?

Probes:

- What was different?
- What happened?
- How did you respond?
- What influenced the outcome?

This question should be used to identify divergent or negative cases rather than assuming that all experiences follow the same pattern.

I. When a Shift Change Cannot Be Arranged

I1. Unsuccessful requests

What usually happens when you cannot arrange the shift change or exchange you need?

Probes:

- What do you do next?
- Do you approach other colleagues?
- Do you contact the charge nurse or manager?
- Does the issue return to the formal roster?
- How does this affect you or your work?

J. Perceptions of the Current System

J1. What works well

What, if anything, works well about the way nurses currently arrange shift changes?

Probes:

- What makes it useful?
- What helps nurses manage their schedules?
- What helps the unit function effectively?

J2. What could be improved

What, if anything, could be improved about the current process?

Probes:

- Communication?
- Digital tools?
- Formal rostering?
- Managerial procedures?
- Transparency?
- Flexibility?
- Documentation?

J3. Future systems

If you could change one aspect of how shift changes are managed in your workplace, what would you change and why?

Probes:

- What would the change look like in practice?
- Who would benefit?
- What challenges might the change create?

K. Closing Questions

K1. Additional experience

Is there anything else about your experience of arranging or exchanging shifts that you think is important and that we have not discussed?

K2. Important message

What do you think nurse managers or healthcare organisations should understand about how nurses actually arrange shift changes?

K3. Final reflection

Is there anything you would like to clarify, correct, or add before we finish?

Thank the participant for their time and contribution.

Remind the participant that:

- Their participation was voluntary.
- Their responses will be treated confidentially.
- Identifying information will be removed or generalized.
- They should contact the research team if they have questions or concerns about the study.

L. General Probing Prompts

The interviewer should use probes selectively and naturally rather than asking every probe after each question.

- “Can you tell me more about that?”

- “Can you describe a specific example?”
- “What happened next?”
- “How did you experience that?”
- “What happened at that point?”
- “What influenced your decision?”
- “How did the other person respond?”
- “What made that easier or more difficult?”
- “Did anything change after that?”
- “Was that typical of your experience, or was it unusual?”
- “Can you explain what you mean by that?”
- “Could you describe what that looked like in practice?”
- “How did that affect what you did next?”
- “What happened after the arrangement was made?”

M. Clarification Prompts

When a participant uses an unclear term or assumes that the interviewer understands a local practice:

- “When you say [term], what does that mean in your unit?”
- “Could you help me understand that a little more?”
- “What does that look like in practice?”
- “Can you give me an example?”
- “I want to make sure I have understood you correctly. Are you saying that…?”
- “What happened when that occurred?”

Thank you for your time and participation in the study.

Supplementary File S2: COREQ 32-Item Checklist

Domain 1: Research Team and Reflexivity

Personal Characteristics

#	Item	Guide question	Status	Manuscript location
1	Interviewer/facilitator	Which author(s) conducted the interview?	**Reported**	Section 2.5, Data Collection
2	Credentials	What were the researcher's credentials?	**Reported**	Section 2.5: “held a PhD in Nursing”
3	Occupation	What was their occupation at the time of the study?	**reported**	Section 2.5 identifies the interviewer as a researcher with more than 10 years of professional and research experience; a specific job title is not stated
4	Gender	Was the researcher male or female?	**Reported**	Sections 2.5 and 2.9
5	Experience and training	What experience or training did the researcher have?	**Reported**	Section 2.5: “more than 10 years of professional and research experience”

Relationship with Participants

#	Item	Guide question	Status	Manuscript location
6	Relationship established	Was a relationship established prior to study commencement?	**Reported**	Section 2.5: “no prior personal, clinical, managerial, supervisory, or other professional relationship with the participants”
7	Participant knowledge of the interviewer	What did participants know about the researcher?	**Reported**	Section 2.5: participants were informed of the interviewer's professional background and the study purpose before each interview
8	Interviewer characteristics	What characteristics were reported about the interviewer/facilitator?	**reported**	Section 2.9, Researcher Reflexivity: reflexive notes on nursing background, gender, and interview interactions are described in

				general terms rather than itemized

Domain 2: Study Design

Theoretical Framework

#	Item	Guide question	Status	Manuscript location
9	Methodological orientation and theory	What methodological orientation was stated to underpin the study?	**Reported**	Section 2.1, Study Design: qualitative descriptive design; reflexive thematic analysis (Braun and Clarke)

Participant Selection

#	Item	Guide question	Status	Manuscript location
10	Sampling	How were participants selected?	**Reported**	Section 2.4, Sampling and Recruitment: purposive maximum-variation sampling
11	Method of approach	How were participants approached?	**Reported**	Section 2.4: unit announcements, liaison with nursing administration, participant referral
12	Sample size	How many participants were in the study?	**Reported**	Sections 2.4 and 3.1: 24 participants
13	Non-participation	How many people refused to participate or dropped out? Reasons?	**Reported**	Sections 2.4 and 3.1: 30 approached; 6 declined because of competing work or scheduling commitments

Setting

#	Item	Guide question	Status	Manuscript location
14	Setting of data collection	Where was the data collected?	**Reported**	Section 2.2, Setting and Organizational Context; Section 2.5: online via a secure institutionally approved platform or in private rooms at the hospital
15	Presence of non-participants	Was anyone else present besides the participants and researchers?	**Reported**	Addressed in the manuscript
16	Description of sample	What are the important characteristics of the sample?	**Reported**	Section 3.1 and Table 1: age, gender, qualification, clinical area, experience, role, shift-swap frequency, interview language, and mode

Data Collection

#	Item	Guide question	Status	Manuscript location
17	Interview guide	Were questions, prompts, and guides provided by the authors? Was it pilot tested?	**Reported**	Section 2.6, Interview Guide; piloted with two nurses not included in the final analysis; full guide in Supplementary File S1
18	Repeat interviews	Were repeat interviews carried out?	**Reported**	Addressed in the manuscript
19	Audio/visual recording	Did the research use audio or visual recording to collect the data?	**Reported**	Section 2.5: interviews were audio-recorded with consent
20	Field notes	Were field notes made during and/or after the interview?	**Reported**	Section 2.5: field notes completed after each interview
21	Duration	What was the duration of the interviews?	**Reported**	Section 2.5: approximately 45–90 minutes
22	Data saturation	Was data saturation discussed?	**Reported**	Section 2.4: sample adequacy is addressed via information power; the manuscript explicitly states that no claim of saturation was made (Section 2.10)
23	Transcripts returned	Were transcripts returned to participants for comment and/or correction?	**Reported**	Section 2.10 explicitly notes that no participant-validation claim was made

Domain 3: Analysis and Findings

Data Analysis

#	Item	Guide question	Status	Manuscript location
24	Number of data coders	How many data coders coded the data?	**Reported**	Section 2.8, Data Analysis: three researchers
25	Description of the coding tree	Did authors provide a description of the coding tree?	**Reported**	Section 2.8 describes the coding process and categories addressed; no formal coding-tree diagram is included (summarized in Supplementary File S3)
26	Derivation of themes	Were themes identified in	**Reported**	Section 2.8: inductive coding followed by sensitizing concepts

		advance or derived from the data?		applied later in interpretation, not imposed as predetermined categories
27	Software	What software, if applicable, was used to manage the data?	**Reported**	Section 2.8: NVivo version 14, used as an organizational tool
28	Participant checking	Did participants provide feedback on the findings?	**Reported**	Section 2.10 explicitly notes that no participant-validation claim was made

Reporting

#	Item	Guide question	Status	Manuscript location
29	Quotations presented	Are participant quotations presented to illustrate themes/findings? Was each quotation identified?	**Reported**	Sections 3.2.1–3.2.4: quotations throughout, identified by participant code (N01–N24)
30	Data and findings consistent	Was there consistency between the data presented and the findings?	**Reported**	Section 3.2: interpretive statements are grounded in and cross-checked against the quoted material
31	Clarity of major themes	Are major themes clearly presented in the findings?	**Reported**	Section 3.2 and Table 2: four major themes clearly presented
32	Clarity of minor themes	Is there a description of diverse cases or discussion of minor themes?	**Reported**	Section 3.2.4, Relational Inequalities, and throughout: refusals, obligation, hierarchy, and unequal access are reported as counterpoints to a uniformly positive account

Reference: Tong A, Sainsbury P, Craig J. Consolidated criteria for reporting qualitative research (COREQ): a 32-item checklist for interviews and focus groups. *International Journal of Quality in Health Care*. 2007;19(6):349–357.
